# RNA-Seq Whole Transcriptome Analysis of Bovine Mammary Epithelial Cells in Response to Intracellular *Staphylococcus aureus*

**DOI:** 10.3389/fvets.2020.00642

**Published:** 2020-12-07

**Authors:** Xiaozhou Wang, Feng Su, Xiaohui Yu, Na Geng, Liping Li, Run Wang, Meihua Zhang, Jianzhu Liu, Yongxia Liu, Bo Han

**Affiliations:** ^1^College of Veterinary Medicine, Shandong Agricultural University, Tai‘an, China; ^2^Research Center for Animal Disease Control Engineering, Shandong Agricultural University, Tai‘an, China; ^3^China Animal Health and Epidemiology Center, Qingdao, China; ^4^College of Veterinary Medicine, China Agricultural University, Beijing, China

**Keywords:** *Staphylococcus aureus*, bovine mastitis, bovine mammary epithelial cells, transcriptome, microRNA, LncRNA 3

## Abstract

*Staphylococcus aureus* (*S. aureus*), a common mastitis pathogen widespread in the natural environment of dairy farms, is capable of invading mammary epithelial cells making treatment difficult. However, the mechanism of the response of bovine mammary epithelial cell to *S. aureus* invasion remains elusive. In this study, transcriptomic analysis and bioinformatics tools were applied to explore the differentially expressed RNAs in bovine mammary epithelial cells (bMECs) between the control and *S. aureus*-treated group. A total of 259 differentially expressed mRNAs (DEmRNAs), 27 differentially expressed microRNAs (DEmiRNAs), and 21 differentially expressed long non-coding RNAs (DElncRNAs) were found. These RNAs mainly enrich the inflammatory response, immune response, endocytosis, and cytokine-cytokine receptor interaction. qRT-PCR was used to analyze the quality of the RNA-seq results. In particular, to the defense mechanism of bovine mammary epithelial cells against intracellular *S. aureus*, the PPAR signaling pathway and the genes (ACOX2, CROT, and NUDT12) were found to be up-regulated to promote the production of peroxisomes and ROS, DRAM1 expression was also up-regulated to facilitate the activation of autophagy, indicating that the above mechanisms were involved in the elimination of intracellular *S. aureus* in bovine mammary epithelial cells.

## Introduction

*Staphylococcus aureus* is a frequently isolated pathogen responsible for bovine mastitis (1). This bacterial disease is economically significant in dairy cows, causing considerable economic losses and a series of food safety concerns (2). Despite the tremendous progress in understanding the pathogenesis of bovine mastitis, the disease remains an important issue in the dairy industry worldwide (3, 4).

Related studies have shown that *S. aureus* invades and survives in bovine mammary epithelial cells, in bMECs protected from host defenses, and in the bactericidal effect of some antimicrobials, thus making the treatment of mastitis caused by *S. aureus* difficult and prone to recurrence (5–7). Moreover, although *S. aureus* is resistant to serval antibiotics (8–10). The administration of antibiotics is currently the most common method for treating bovine mastitis (11). Hence, understanding the potential molecular regulatory mechanisms of *S. aureus* invading bovine mammary epithelial cells is particularly important.

MicroRNAs (miRNAs) are endogenous small non-coding RNAs (22–25 nucleotides) universally expressed in higher eukaryotic cells that play a crucial role in post-transcriptional gene regulation (12). Previous research has shown microRNAs as a vital part of the mammalian host response to bacterial infection, involved in the host immune response (13–16). Long non-coding RNAs (lncRNAs) are a class of non-coding RNAs over 200 nucleotides in length that are essential regulators of the immune response. Several researchers have reported the specific involvement of lncRNAs in the response of host cells to bacterial infection (17, 18). Their role in regulating gene-encoding products involved in the immune response, including direct interactions with chromatin, RNA, and proteins has been one of the most recent discoveries (19, 20). In general, the characterization of RNA regulatory networks represents a new area in the field of host-pathogen interactions.

High-throughput transcriptome sequencing has effectively been used to explore candidate mRNAs, lncRNAs, and miRNAs with a differential expression that may be involved in specific biological processes (21–23). Thus, it laid the foundation for subsequent integration of whole transcriptome analysis. To date, several studies have focused on the bovine mammary tissue or epithelial cells transcriptional response to *S. aureus* showing significant changes in gene expression following *S. aureus* infection (24–28). However, complete transcriptome analysis of the response of bovine mammary epithelial cells to intracellular *S. aureus* has not been reported. This study, for the first time, details the interpretations of whole transcriptome bMECs infected with intracellular *S. aureus*.

The purpose of this study was to explore the transcriptional regulation of bovine mammary epithelial cells after *S. aureus* invasion and identify related candidate mRNAs, lncRNAs, and miRNAs. Finally, the possible functions of the identified RNAs were also discussed. These findings provide a base for the study on the pathogenic mechanism of intracellular *S. aureus* and offer several potential targets for the treatment of *S. aureus*-mastitis.

## Materials and Methods

### Bacterial Strains and Growth Conditions

*Staphylococcus aureus* strain ATCC25923 was inoculated on a Luria-Bertani (LB) Agar and incubated at 37°C for 24 h. A single colony was randomly selected and cultured in LB broth with agitation at 37°C for 12 h and the growth was monitored by measuring the OD_600nm_.

### Cell Culture

MAC-T cells are sensitive to the regulation of the extracellular matrix and lactogen. MAC-T cells after differentiation can synthesize and secrete α- and β-casein. It represents an *in vitro* model of bovine lactation (29). Bovine mammary epithelial cell line MAC-T cells were cultured in a cell culture plate with a growth medium consisting of Dulbecco's modified eagle culture medium (DMEM) with 10%(v/v) FBS and maintained in 5% CO_2_ at 37°C. Cells that cultured to monolayer confluence were used for further experiments.

### Intracellular Infection Model

The model of intracellular infection was established in our previous study (30). In brief, MAC-T cells were seeded on 6-well cell plates, and when cells were cultured to monolayers, *S. aureus* (OD_600nm_ = 0.8–1.2) was inoculated with (treatment group) or without (control group) an MOI of 8:1. After 2 h of incubation, the cells were washed three times in PBS. The cells from the treatment and control groups were further placed into fresh medium supplemented with lysostaphin (10 μg/mL) and gentamicin (50 μg/mL) to kill extracellular bacteria. After 12 min, extracellular fluid was collected for plate culture experiments to verify that extracellular bacteria were eliminated. The cells were again washed three times with PBS to remove extracellular bacteria and dead cells (30), and incubated for 2 h in 10% FBS-DMEM.

### RNA Extraction and cDNA Library Construction

The total RNA from the control and *S. aureus*-treated group (three samples per group) were extracted using RNAiso Plus (Takara, Dalian, China) according to the manufacturer's instructions. Qualitative and quantitative total RNA was quantified using Nano Drop and the Agilent 2100 Bioanalyzer (Thermo Fisher Scientific, MA, USA). These RNAs were divided into two parts for library construction of RNA or small RNA, respectively.

Total RNA was treated with DNase I which degraded double-stranded and single-stranded DNA and ribosomal RNA (rRNA) was removed using the Ribo-Zero™ rRNA Removal Kit (Illumina, San Diego, CA, USA). Fragmentation of the purified RNA was carried out at a specific temperature and ionic environment. The first strand cDNA was produced by PCR in the first strand reaction system, along with the second-strand cDNA. A-Tailing Mix and RNA Index Adapters were added and incubated to carry out end repair, and purification was performed using Ampure XP Beads. Distribution of fragment sizes in the sequencing database was examined using the Agilent 2100 Bioanalyzer, and the libraries were quantified using qRT-PCR (TaqMan Probe). Finally, the qualified libraries were pair-end sequenced on the Illumina Hiseq 4000 platform (BGI, Shenzhen, China).

### Quality Control of Raw Data

Before alignment, FastQC was used to check the quality of the raw reads generated by the Illumina Hiseq 4000 platform that were filtered by removing the dirty raw reads. Reads containing adapter, an unknown base >10%, and low-quality reads (The base number of threshold mass ≤10 accounts for more than 50% of the total reading) were removed to obtain clean reads of mRNA. PolyA, adapter, low-quality, and length <18 nt tags were removed to get clean reads of miRNAs.

### Reads Alignment and Differential Expression Analysis of RNA-Seq

The transcriptome data were submitted to the Sequence Read Archive (SRA) of the National Center for Biotechnology Information (NCBI) (https://www.ncbi.nlm.nih.gov/sra/), with the BioProject ID, PRJNA591729. In this study, gene model annotations and reference genomes (ARS-UCD1.2/bosTau9) were accessed from UCSC (31), lncRNA model annotations were accessed from NONCODE (32), and miRNA model annotations were obtained from miRbase (33).

The clean reads of each sample were mapped using HISAT2 for mRNA and lncRNA to the index from the reference genome. StringTie was used to assemble transcripts for each sample to obtain all assemblies. After initial assembly, assembled transcripts were merged by the StringTie merge module, creating a uniform set of transcripts for all samples. StringTie was run again by the merge function to re-calculate the abundances of the merged transcripts after merging all assemblies and generated read coverage tables. StringTie also provided a Python script to calculate the count matrix of mRNA or lncRNA directly from the file created in the previous step (34).

Conversely, Bowtie was applied for miRNA to map the clean reads of each sample to the index from the reference genome (35). miRDeep2 calculated the count matrix of each miRNA for each sample (36).

The DESeq2 package in R identified the count matrix between the control and S. aureus-treated group, and the results according to the *P*-values (37). The pheatmap in R were used for clustering. lncRNAs, miRNAs, and mRNAs with a *P* < 0.05 and | log2(fold-change) | > 1 were set as the threshold for being differentially expressed.

### Protein–Protein Interaction (PPI) Analysis

Understanding all functional interactions between expressed proteins is essential for a system-wide understanding of cellular function. The STRING database aims to consolidate known and predicted protein–protein association data. Network analysis of DEmRNAs protein-protein interactions were obtained using the STRING database (38), and Cytoscape was used to draw the PPI network.

### Prediction of the Target Gene of lncRNAs and miRNAs

The target genes of the lncRNAs were identified using a large-scale RNA-RNA interaction prediction tool, RIsearch2 (39). The target genes of the miRNAs were predicted using TargetScan and miRWalk (40, 41).

### GO Annotation and KEGG Pathway Analysis of DEmRNAs and Target Genes of DEmiRNAs and DElncRNAs

Gene Ontology (GO) enrichment analysis was performed using the DAVID 6.8 functional annotation tool. The Kyoto Encyclopedia of Genes and Genomes (KEGG) pathways were analyzed by the clusterProfiler R package (42, 43).

### Competitive Endogenous RNAs (ceRNAs) Analysis of DEmRNAs, DEmiRNAs, and DElncRNAs

DEmRNAs, DEmiRNAs, and DElncRNAs crosstalk networks were constructed based on the hypothesis of competitive endogenous RNA (ceRNA) (44). ceRNA act as a sponge for microRNA (miRNA) through its binding site, and changes in ceRNA abundance in individual genes can regulate miRNA activity (45). Thus, understanding this new RNA crosstalk could provide insight into gene regulatory networks. Correlation analysis was performed in R (*P* < 0.05 & cor < − 0.4) and the networks were constructed by cytoscape software (46).

### qRT-PCR Identification of Differentially Expressed mRNAs

Randomly selected five (PIDD1, TNFRSF18, DEPDC5, TMEM102, TRIM32) and three (CD36, DRAM1, CXCR1) genes of biological interest from DEmRNAs were tested by qRT-PCR to verify the reproducibility and repeatability of the differentially expressed genes in RNA-Seq data. Total RNA was extracted using RNAiso Plus (Takara, Dalian, China) and reverse-transcribed using the PrimeScript® RT regent kit with gDNA Eraser (Perfect Real Time) (Takara, Dalian, China) according to the manufacturer's instructions. Quantitative PCR analysis was performed using the LightCycler® 96 (Roche Diagnostics GmbH, Germany) with TB Green™ Premix Ex Taq™ II (Tli RNaseH Plus) Kit (Takara, Dalian, China) following the manufacturer's instructions. The reaction mixtures in a 96-well plate were run at 95°C for 120s, followed by 40 cycles at 95°C for 15 s and 65°C for 30 s. qRT-PCR was performed in triplicate for each cDNA sample to ensure repeatability. The glyceraldehyde-3-phosphate dehydrogenase (GAPDH), actin beta (ACTB), and peptidylprolyl isomerase A (PPIA) genes were set as endogenous reference genes. All primers used in this study are listed in Table 1.

**Table 1:** Primers used in this study.

**Gene**	**Primer sequence (5^′^ → 3^′^)**	**Gene ID**	**Amplicon size (bp)**
PIDD1-F	TGCATTGGGCCCTGATCTC	100137737	89
PIDD1-R	CCGTCCTGCACACGACTGTA		
DRAM1-F	GTGTCCTGCGCAGCTGTCATA	533992	128
DRAM1-R	ACTGTCCATTCACAGATCGCACTC		
TNFRSF18-F	TGTATCCAGCCCGAGTTCCAC	516256	131
TNFRSF18-R	ACGGCACAGTCAACACACTCAA		
DEPDC5-F	GTGCGACTGGAACAGGCAGA	521542	84
DEPDC5-R	CAGGTTGATGGCCTCCAGGTA		
TMEM102-F	GAACTAACCCAGCTGATCCAGGAG	508014	126
TMEM102-R	CCTGCGATGAATGAGACTAAGCAA		
TRIM32-F	GCGGCAACTACCGCATACAA	521975	96
TRIM32-R	AGAAGCTCAGCACAAAGCTATCCA		
CD36-F	TTGATGTGCAGAATCCAGATGAAG	281052	183
CD36-R	CAACTGATAGCGAGGGTTCAAAGA		
GAPDH-F	GATGGTGAAGGTCGGAGTGAAC	281181	100
GAPDH-R	GTCATTGATGGCGACGATGT		
CXCR1-F	TCCCTGTGAGATAAGCACTGAGACAC	281863	118
CXCR1-R	GCTGTATAAGATGACCAGCATCACCA		
ACTB-F	CATCGGCAATGAGCGGTTC	280979	152
ACTB-R	ACAGCACCGTGTTGGCGTAG		
PPIA-F	GGTGGTGACTTCACACGCCATA	281418	100
PPIA-R	TGCCAGGACCTGTATGCTTCAA		

### Statistical Analysis

The relative fold change of target gene expression was calculated by the 2^−ΔΔ*Ct*^ method (47), and a Students *t*-test examined significant differences between conditions. ΔCt value was determined by subtracting the target Ct of each sample from the GAPDH, ACTB, and PPIA Ct values.

## Results

### Transcriptome Assembly Profiles Evaluation

The quality of six mRNA and lncRNA transcriptome sequence expression profiles of mammary epithelial cells are summarized in Table 2. Clean data were obtained after filtration from downstream analysis.

**Table 2:** mRNA and lncRNA sequence quality.

**Sample**	**C1**	**C2**	**C3**	**T1**	**T2**	**T3**
**Group**	**Control**	**Control**	**Control**	**Treatment**	**Treatment**	**Treatment**
Total raw reads	135018358	133373384	133372480	133374488	133373544	133373594
Total clean reads	127339136	126512562	126149020	126426654	125894398	125895540
Total clean base	12733913600	12651256200	12614902000	12642665400	12589439800	12589554000
Clean reads ratio	94.312%	94.856%	94.584%	94.791%	94.392%	94.393%

### Analysis of Differentially Expressed mRNAs

**Figure 1:**
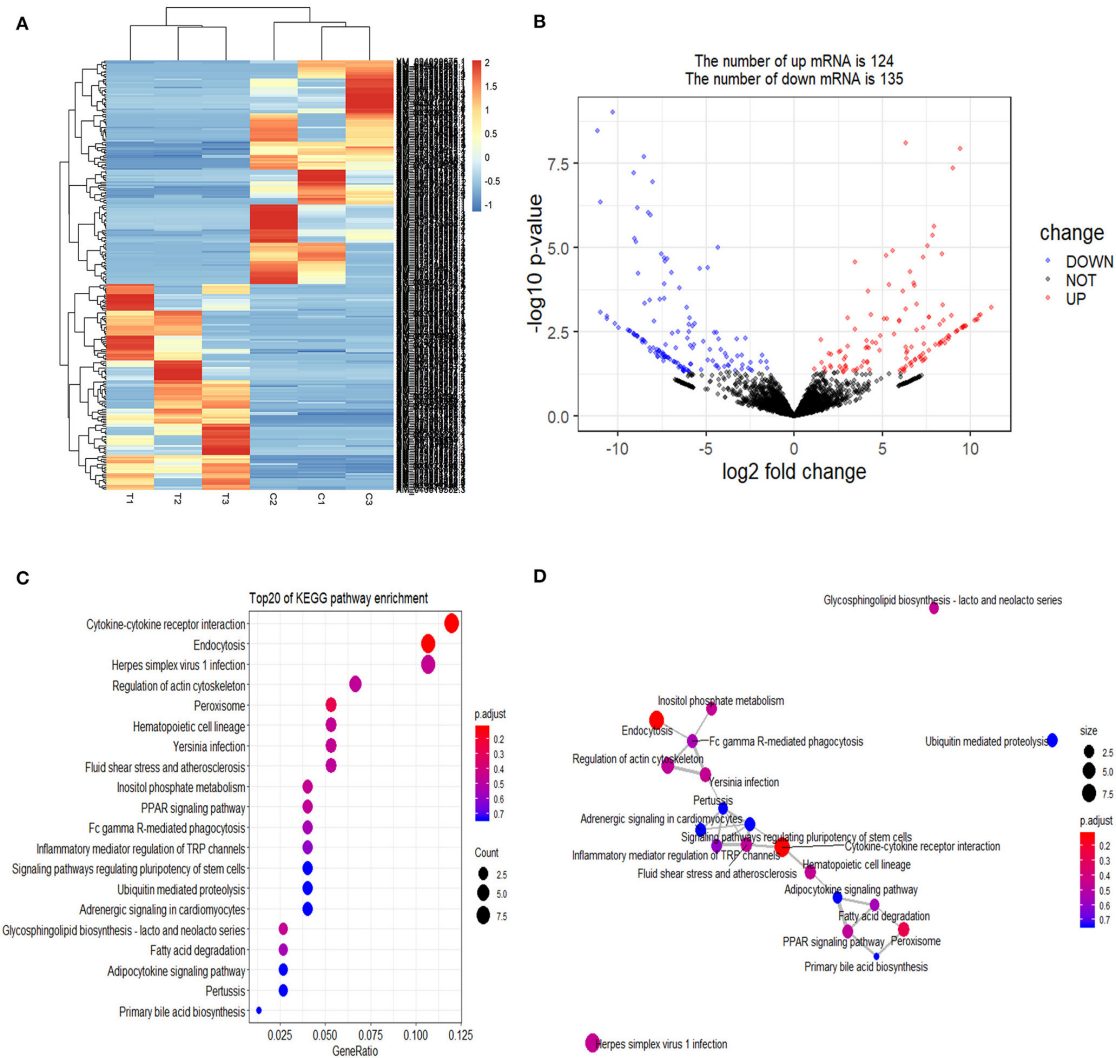
Screening and enrichment analysis of differently expressed mRNAs (DEmRNAs) in *S. aureus* infected mammary epithelial cells compared with non-infected mammary epithelial cells. **(A)** Cluster analysis of DEmRNAs in mammary epithelial cells between the control group (C1, C2, and C3) and treatment group (T1, T2, and T3). Red indicates highly expressed genes, and blue indicates low expressed genes. Each column represents a sample, and each row represents a gene. On the left is the tree diagram of mRNA clustering, and on the right is the name of each mRNA. The closer the two mRNA branches are, the closer their expression level is. The upper part is the tree diagram of sample clustering, and the bottom is the name of each sample. The closer the two-sample branches are to each other, the closer the expression pattern of all genes in the two samples are and the trend of the more recent gene expression. **(B)** Volcano plot of global DEmRNAs in mammary epithelial cells between the control group and treatment group. Red dots (Up) represent significantly up-regulated genes [*P* < 0.05, | log2(fold-change) | >1]; blue dots (Down) represent significantly down-regulated genes [*P* < 0.05, log2(fold-change) < −1]; black dots represent insignificantly differential expressed genes. **(C)** KEGG pathway classified annotation of DEmRNAs in mammary epithelial cells. The pathway is exhibited in the left axis, and the size of the circle represents the number of genes listed in the right axis. **(D)** The relationship between these pathways is illustrated.

Cluster pattern analysis of DEmRNAs between the control (*n* = 3) and *S. aureus*-treated groups (*n* = 3) is illustrated in Figure 1A. A total of 259 DEmRNAs (Supplementary File 1) were filtered by the thresholds of *P* < 0.05 and | log2(fold-change) | > 1, out of which 124 were up-regulated and 135 were down-regulated (Figure 1B).

**Figure 2:**
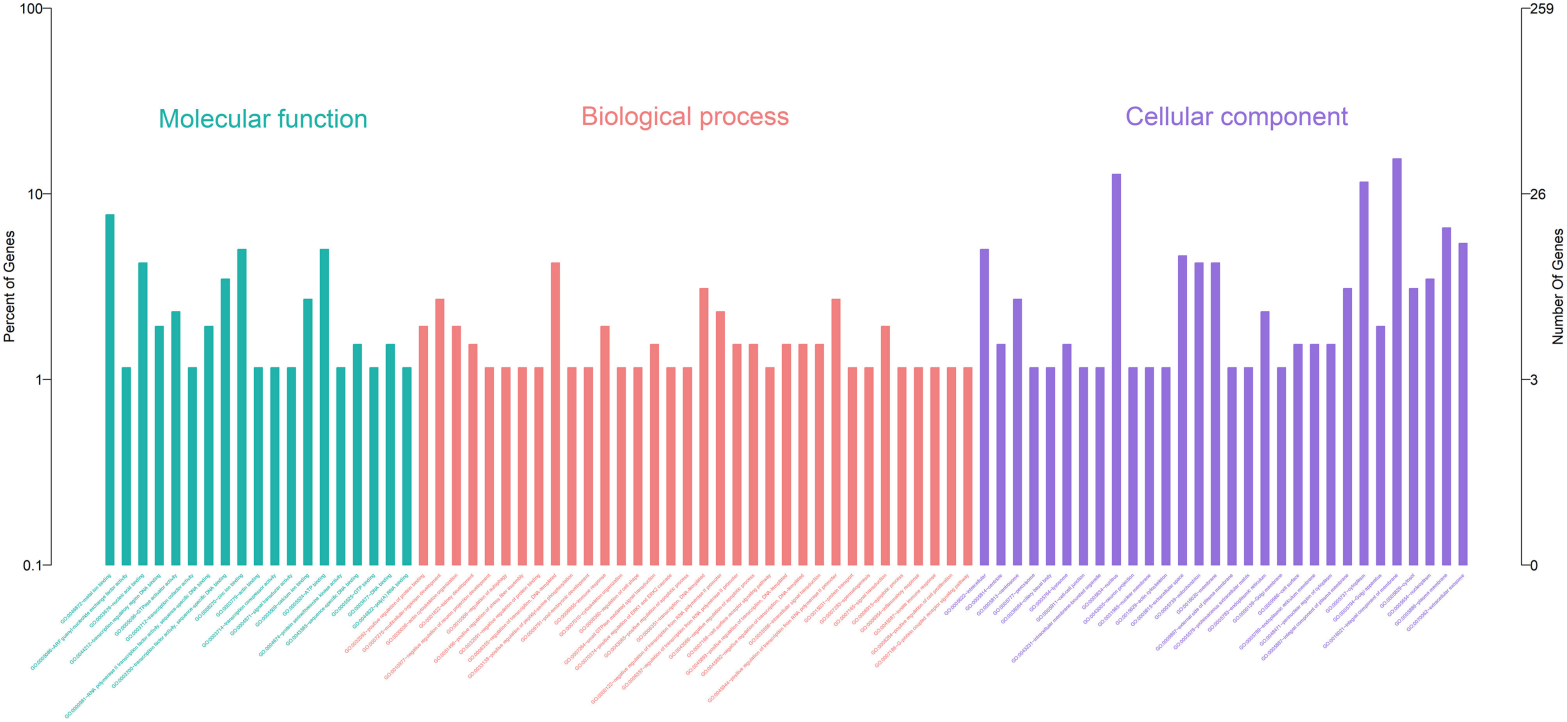
Annotation of DEmRNAs using Gene Ontology (GO) in mammary epithelial cells. The number of mRNAs for each GO annotation is exhibited in the right axis, and the proportion of genes for each GO annotation is listed on the left axis.

Gene Ontology (GO) enrichment analysis showed that 259 DEmRNAs were distributed into 83 GO terms (Supplementary File 2) and divided into three categories based on molecular function, biological process, and cellular components (Figure 2). The top 10 enrichment GO terms, such as the metal ion binding, integral component of the membrane, extracellular exosome, intracellular, and extracellular space were also significantly enriched.

The enrichment analysis of the KEGG pathway in DEmRNAs is depicted in Figure 1C. The top 20 enriched pathways included “Endocytosis,” “Cytokine-cytokine receptor interaction,” and “PPAR signaling pathway,” which may be associated with inflammation development. The relationship between these pathways is reflected in Figure 1D.

**Figure 3:**
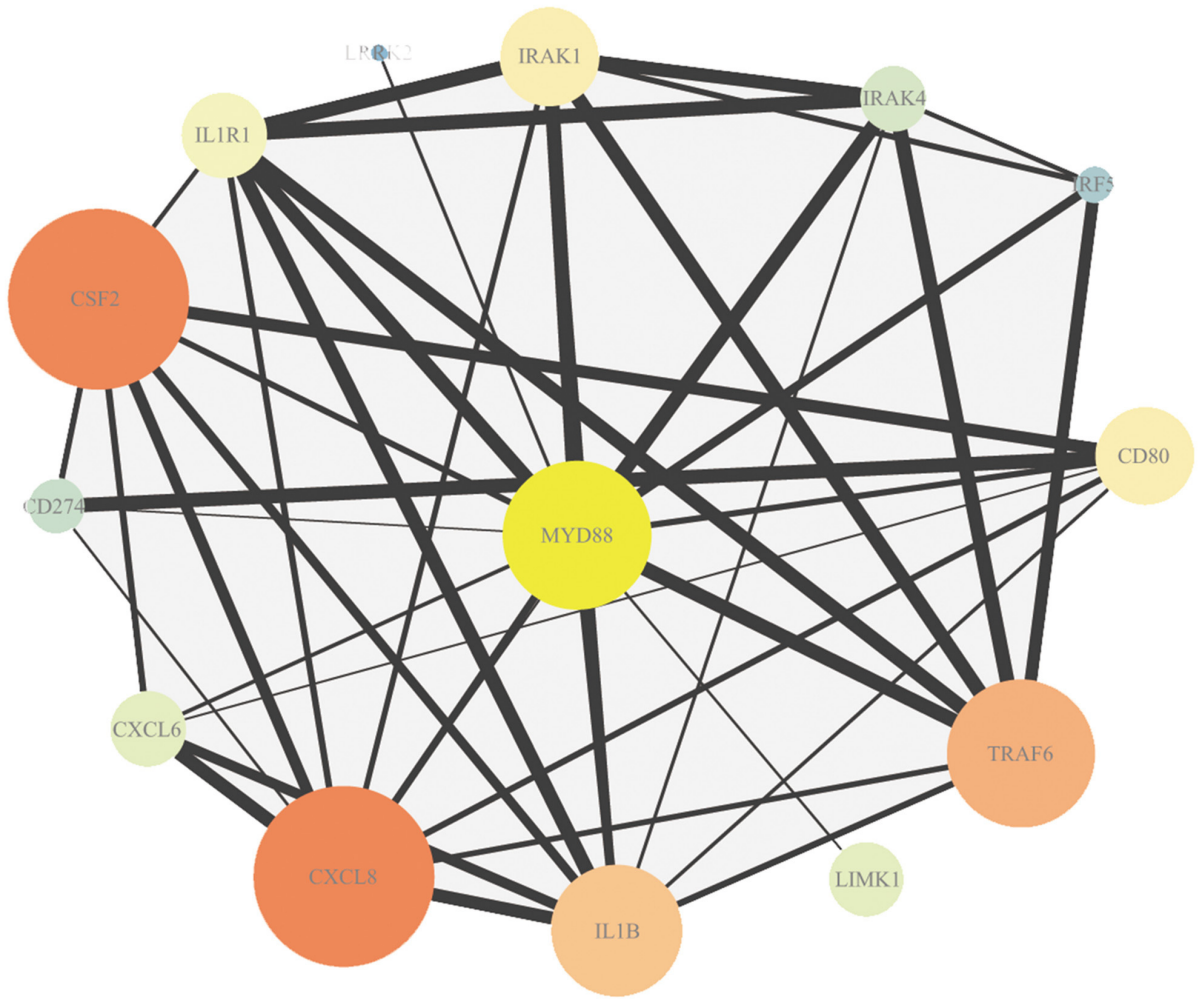
Protein–protein interaction (PPI) analysis of DEmRNAs. Map node size and color to degree, low values to small sizes and dark colors. Map edge size to the combined source, low values to small sizes.

The association in STRING includes direct (physical) and indirect (functional) interactions. The PPI network (Figure 3) showed that myeloid differentiation-factor 88 (MyD88) is a central protein and interacts with multiple proteins.

### Analysis of Differentially Expressed lncRNA

**Figure 4:**
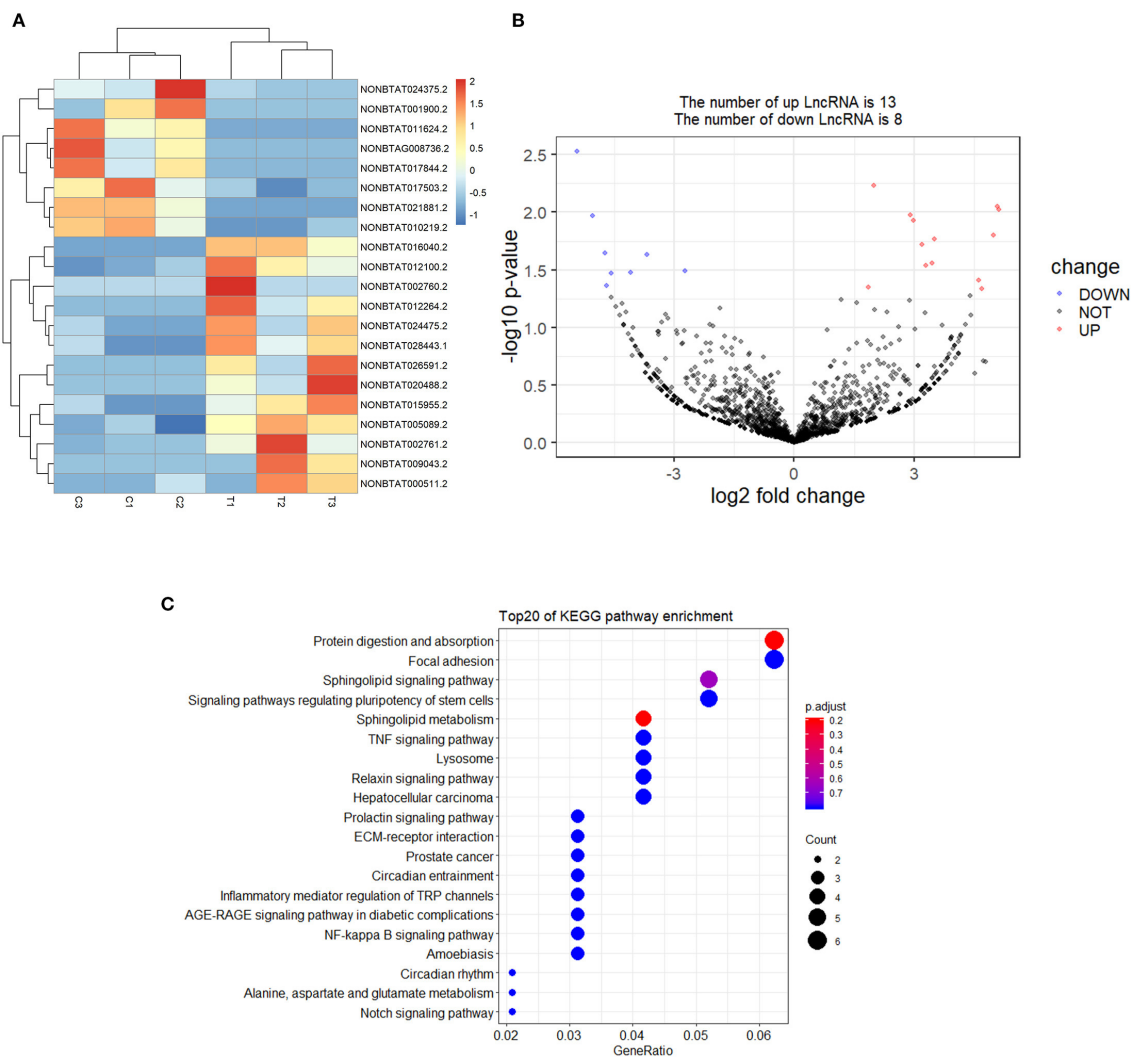
Screening and enrichment analysis of differently expressed lncRNAs (DElncRNAs) in *S. aureus* infected mammary epithelial cells compared with non-infected mammary epithelial cells. **(A)** Cluster analysis of DElncRNAs in mammary epithelial cells between the control group and treatment group. **(B)** Volcano plot of global DElncRNAs in mammary epithelial cells between control group and treatment group. **(C)** KEGG pathway classification for the annotation of DElncRNAs in mammary epithelial cells.

**Figure 5:**
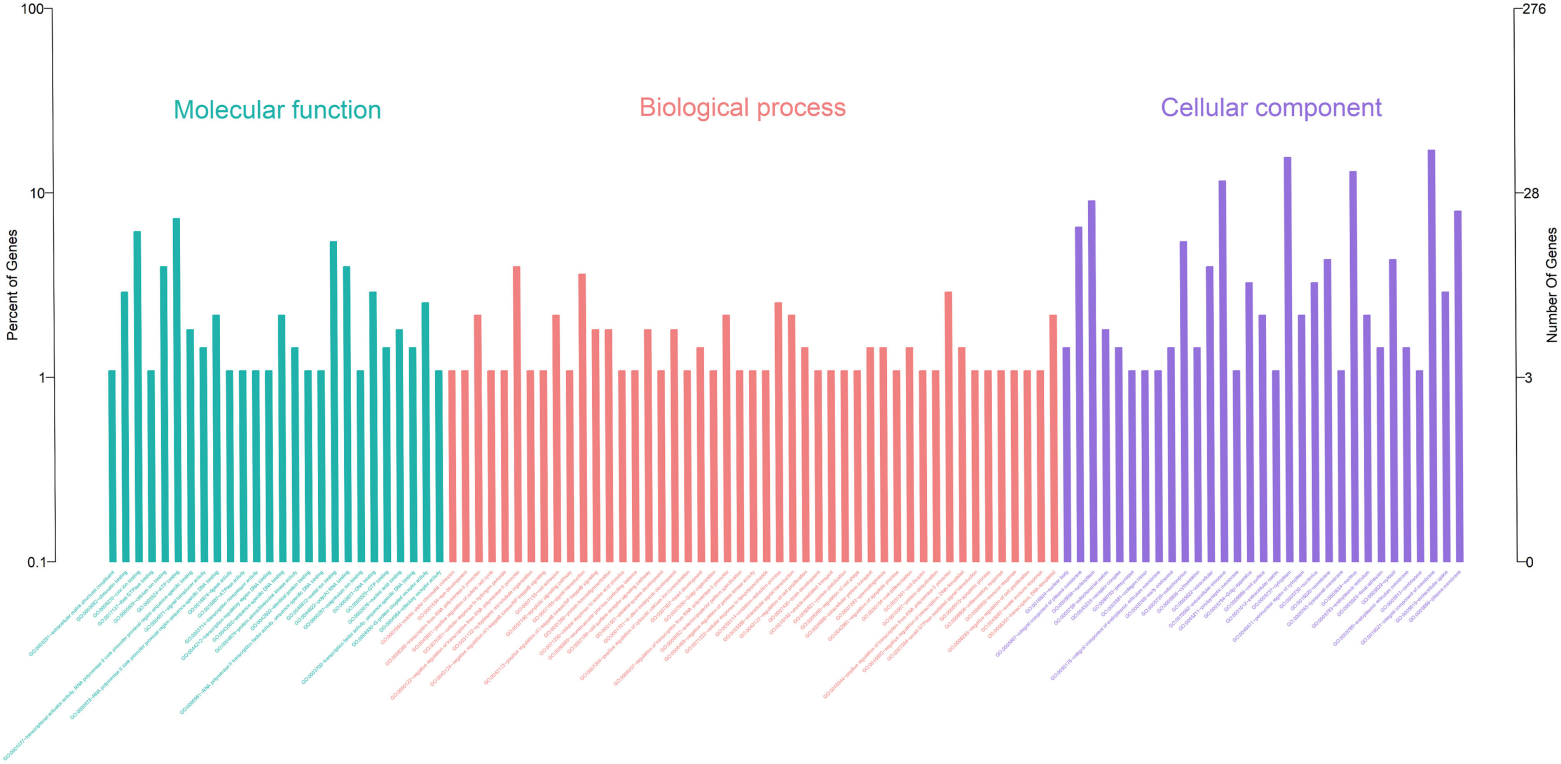
Annotation of DElncRNAs using Gene Ontology (GO) in the mammary epithelial cells.

A total of 21 DElncRNAs were obtained (Supplementary File 3), of which 13 were up-regulated and 8 were down-regulated (*P* < 0.05 and | log2(fold-change) | > 1). Volcano plot showed the DElncRNAs between the control group and treatment groups (Figure 4A). The heatmap showed hierarchical clustering for DElncRNAs in Figure 4B. A total of 288 DElncRNA target genes were predicted. Figure 5 depicts 107 GO terms (Supplementary File 4) from the GO analysis. The “integral component of the membrane” term was the found to be most significant enrichment. Figure 4C depicts the enrichment analysis of the KEGG pathway in DElncRNA targets. Figure 8A shows two common target genes (Supplementary File 5) from DEmRNA and DElncRNA targets and KEGG enrichment analysis of these genes is shown in Figure 8C.

### Summary of miRNA Sequencing

The quality of six miRNA transcriptome sequence expression profiles of mammary epithelial cells is presented in Table 3. The data used for downstream analysis was filtered.

**Table 3:** miRNA sequence quality.

**Sample**	**C1**	**C2**	**C3**	**T1**	**T2**	**T3**
**Group**	**Control**	**Control**	**Control**	**Treatment**	**Treatment**	**Treatment**
Raw tag count	13935997	13916721	14223015	14965296	14386800	14745979
Clean tag count	12589674	12995887	13399505	14374700	13798302	14033353
Percentage (%)	90.34	94.38	94.21	96.05	95.91	95.17

**Figure 6:**
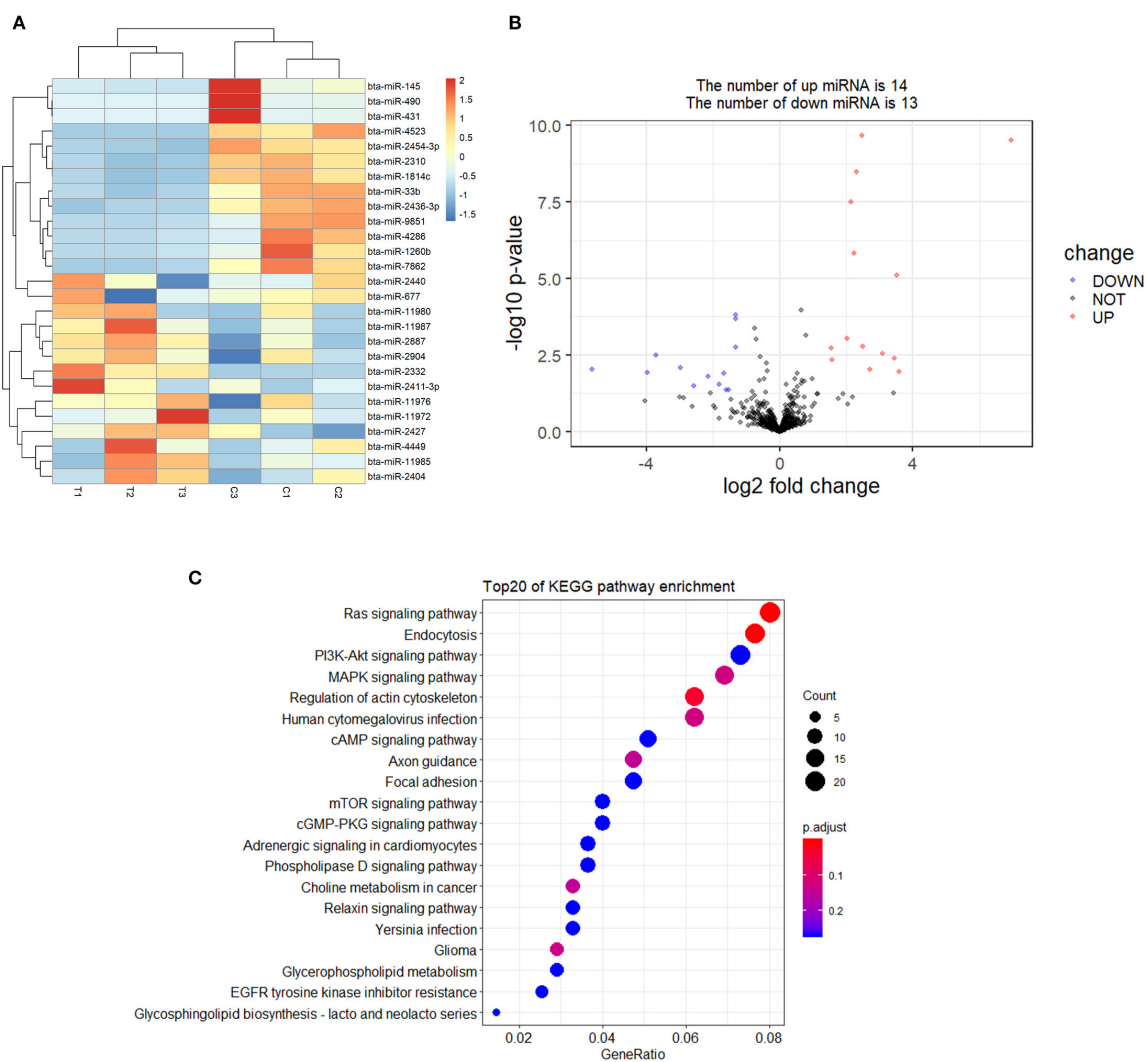
Screening and enrichment analysis of differently expressed miRNAs (DEmiRNAs) in *S. aureus* infected mammary epithelial cells compared with non-infected mammary epithelial cells. **(A)** Cluster analysis of DEmiRNAs in mammary epithelial cells between the control group and treatment group. **(B)** Volcano plot of global DEmiRNAs in mammary epithelial cells between the control group and treatment group. **(C)** KEGG pathway classified annotation of DEmiRNAs in mammary epithelial cells.

**Figure 7:**
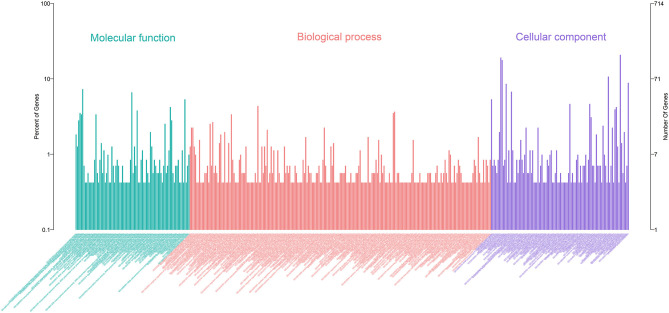
Annotation of DEmiRNAs using Gene Ontology (GO) in mammary epithelial cells.

**Figure 8:**
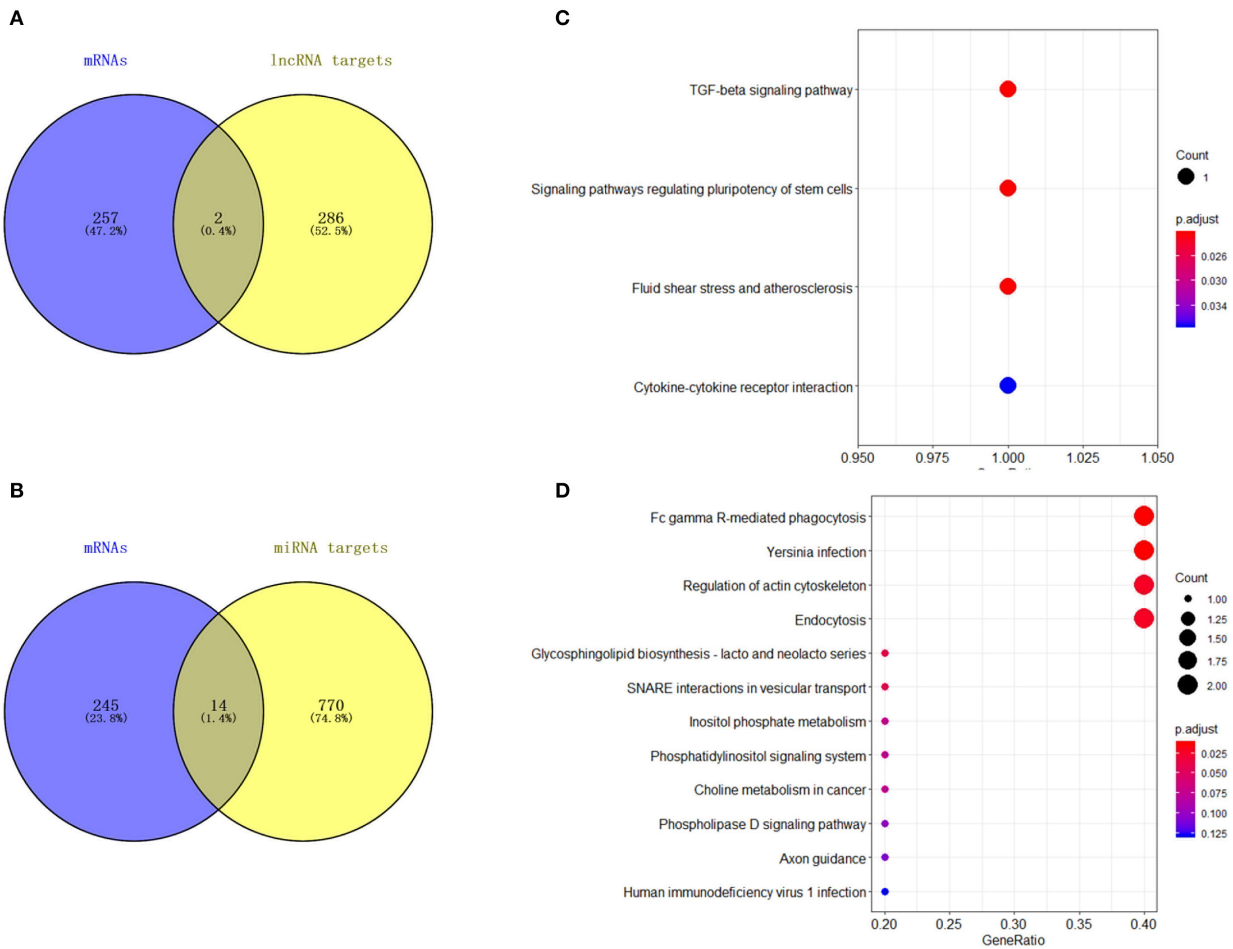
Analysis of DElncRNA target genes and DEmiRNA target genes. **(A)** Venn map analysis of DElncRNA target genes and DEmRNAs**. (B)** Venn map analysis of DEmiRNA target genes and DEmRNAs. **(C)** KEGG pathway classification for the annotation of two common target genes from the DEmRNAs and DElncRNA target genes in mammary epithelial cells. **(D)** KEGG pathway classification for the annotation of 14 common target genes from the DEmRNAs and DEmiRNA target genes in mammary epithelial cells.

A total of 27 DEmiRNAs were identified (*P* < 0.05 and | log2(fold-change) | > 1) (Supplementary File 6) and the volcano plot also shows these DEmiRNAs (Figure 6B). The heatmap of the DEGs shows clustering of DEmiRNAs (Figure 6A). Figure 7 shows the predicted 784 target genes of DEmiRNAs that enriched the 417 GO terms (Supplementary File 7). KEGG pathway enrichment analysis of DEmiRNAs is shown in Figure 6C, suggesting DEmiRNAs major role in the “Ras signaling pathway,” “Endocytosis,” and “PI3K-AKT signaling pathway”. There were 14 common target genes (BASP1, RAB11FIP4, PIP5K1C, VAMP4, DHRS12, LBH, MOB3B, ABO, FOXQ1, WDFY2, DYRK1B, NCKAP5L, TMEM59L, LIMK1) from DEmRNA and DEmiRNA targets Figure 8B. Figure 8D shows KEGG enrichment analysis of DEmRNA and DEmiRNA targets.

### Competitive Endogenous RNAs (ceRNAs) Analysis

**Figure 9:**
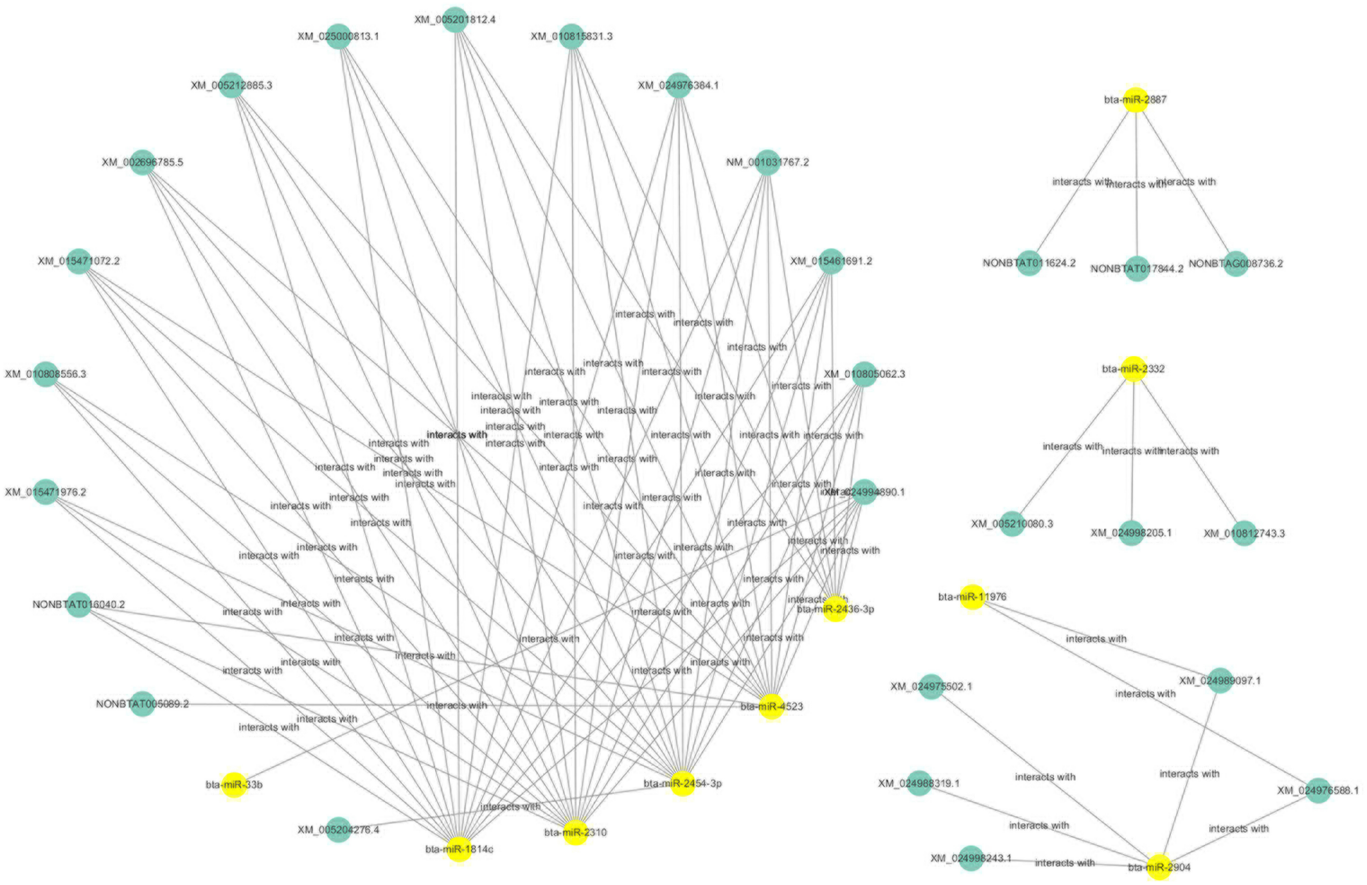
Competitive endogenous relationships between DEmRNAs, DEmiRNAs, and DElncRNAs are shown.

The ceRNAs network is shown in Figure 9 which reflects the competitive endogenous relationships between DEmRNAs, DEmiRNAs, and DElncRNAs.

### Confirmation of Gene Expression With qRT-PCR

**Figure 10:**
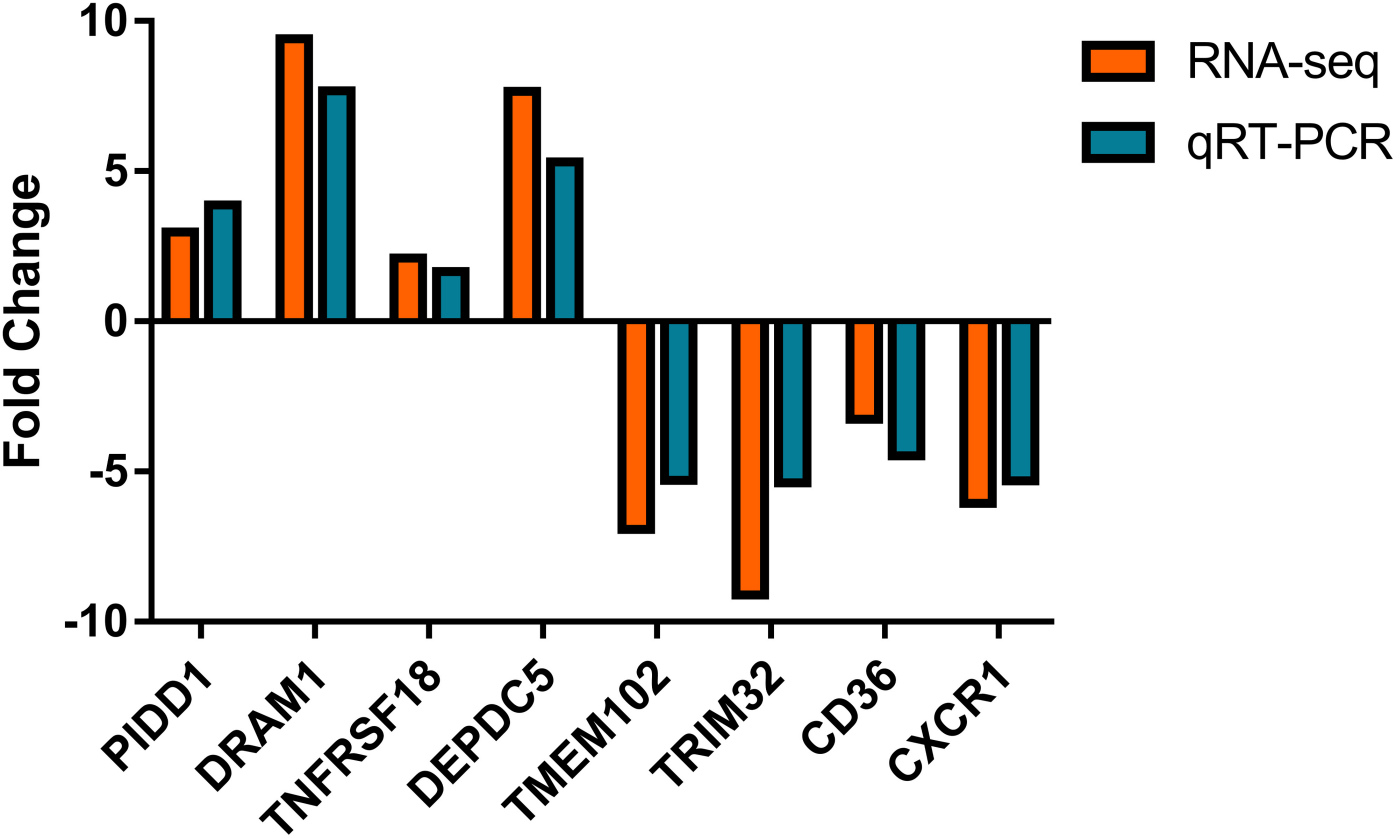
Expression levels of selected DEGs quantified by quantitative reverse transcription-PCR (qRT-PCR). GAPDH, ACTB, and PPIA were used as an internal control, and data are presented as fold change (*N* = 3 per group).

Nine differential expressed genes (PIDD1, DRAM1, TNFRSF18, DEPDC5, TMEM102, TRIM32, CD36, CXCR1) were randomly selected and quantified using qRT-PCR to confirm differentially expressed genes in mammary epithelial cells obtained by RNA-seq between the control and treatment group. The gene symbol corresponding to all mRNAs is shown in Supplementary File 8. PIDD1, DRAM1, TNFRSF18, and DEPDC5 were the up-regulating genes while TMEM102, TRIM32, TNFRSF25, CD36, and CXCR1 were down-regulating in the high-throughput RNA-Seq. The expression of these genes was confirmed by qRT-PCR (Figure 10).

## Discussion

*Staphylococcus aureus* often causes subclinical bovine mastitis and persistent intramammary infections. Ineffective pathogen clearance often leads to chronic and persistent infections (7). Studies have shown that *S. aureus* invades udder epithelial cells, which protects the pathogen from host defenses and antibiotics (6). Thus, it is particularly important to understand the response associated with bovine mastitis at a molecular level. In this study, we performed a comprehensive assessment of the whole transcriptomic profile of bMECs infected by *S. aureus* intracellularly, it contributed to a more embedded understanding of the transcriptome regulation behind this biological process.

Wang et al. investigated the transcriptional responses of primary bovine mammary epithelial cells against three S. aureus strains with different virulent factors using a tag-based high-throughput transcriptome sequencing technique (24). Li et al. identified functional miRNAs in bovine mammary glands infected with *S. aureus* on Chinese Holstein cows using Solexa sequencing and bioinformatics (25). Fang et al. compared the expression of mRNAs and miRNAs at after 24 h of intra-mammary infection (IMI) with high or low concentrations of *S. aureus* (26). The current study mainly focused on the defense mechanism of bMECs against intracellular *S. aureus*. Differences were observed from the previous research from the experimental designs to the conclusion.

Through the GO functional enrichment analysis for DEmRNAs, functional molecular processes were found to be related to the inflammatory response, immune response, peroxisome, regulation of autophagy, and positive regulation of ERK1 and ERK2 cascade. Related research showed that peroxisomes play an indispensable role in the generation of reactive oxygen species (ROS) (48). ROS is crucial in the signaling and defense of biological organisms and can also contribute to bacterial killing (49–51). Previous studies showed that *S. aureus* induces bMECs triggering immune responses and ROS production (52–54). Autophagy is a highly conserved mechanism that maintains homeostasis by removing damaged organelles and cytoplasmic proteins. It is also an immune response pathway that can eliminate intracellular bacteria (55–57). Bacterial infection induces autophagy and transports bacteria to the lysosome for degradation by autophagosomes (58, 59). The ERK1/2 signaling pathway has been shown to respond to the autophagy regulation of intracellular pathogens (60). KEGG pathway analysis identified several critical pathways, such as endocytosis, cytokine-cytokine receptor interaction, PPAR signaling pathway, and the inflammation mediator regulation of TRP channels. Endocytosis is essential for the entry of pathogens into cells and followed by its replication. Bacteria use this mechanism to utilize the osmotic network of the endocytic organelles to enter the cytoplasmic or replicating site and reach the relevant intracellular compartment (61, 62). Cytokine-cytokine receptor interaction is also reported to be involved in clinical mastitis (63). Peroxisome proliferator-activated receptor (PPAR) has anti-inflammatory effects inevitably linked to inflammation (64). Studies have shown that transient receptor potential (TRP) channels are associated with various factors and mechanisms that can activate/modulate inflammation through innate immunity (65, 66). They also play a key role in *S. aureus* elimination from bovine mammary epithelial cells.

In this study, some genes were up-regulated in the *S. aureus*-treated bMECs, such as ACOX2 (acyl-CoA oxidase 2), CROT (carnitine O-octanoyltransferase), NUDT12 (nudix hydrolase 12), and DRAM1 (DNA damage regulated autophagy modulator 1). Among these, three genes (ACOX2, CROT, and NUDT12) were enriched in peroxisomes. Additionally, peroxisomes play an integral role in the production of ROS. ROS levels acutely increased during cellular stress and the process of bacterial killing. Many studies supported the indispensable role of cellular stressors in regulating the innate immune responses (51). A new study reported that ROS can enhance macrophage antimicrobial activity against intracellular *S. aureus* (67). In the *S. aureus* treated group, DRAM1 expression was up-regulated. Moreover, in the case of DRAM1 over-expression, autophagosome production could be triggered by enriching DRAM1 on the Golgi membrane (68). Besides, DRAM1 can affect autophagy by the acidification of lysosomes, the fusion of lysosomes with autophagosomes, and autophagosome clearance (69). However, although autophagy has an antibacterial effect, there is evidence that pathogens have developed several ways to evade this mechanism (70). *S. aureus* can avoid autophagy clearance in bMECs by impairing lysosome function (30). Which may be an important mechanism of *S. aureus* to evade the host's immune response.

Conversely, certain genes including CD36 (CD36 molecule), CXCR1 (chemokine C-X-C motif receptor 1), BMP4 (bone morphogenetic protein 4), and TNFRSF25 (TNF receptor superfamily member 25) were down-regulated in *S. aureus*-treated bMECs. CD36 is a transmembrane glycoprotein receptor and contributes to the host's innate defense against *S. aureus*. It binds to TLR2 (toll like receptor 2) and recognizes the *S. aureus* cell wall diacylglycerol, inducing phagocytosis and cytokine production. In addition, CD36 expression on macrophages plays a significant role in host control of inflammation and skin damage during skin infection caused by *S. aureus* (71). Previous studies showed that blocking cell surface CXCR1 expression could be used as a beneficial treatment against *S. aureus* infection by disrupting the balance between inflammation and bacterial clearance (72). BMP4 facilitates leukocyte recruitment and inflammation improvement (73). The down-regulation of CD36, CXCR1, and BMP4 expression is not conducive to eliminating the bacteria but may be beneficial for *S. aureus* to evade its destruction.

Analysis of DElncRNA target profiles revealed that DElncRNAs participate in the regulation of the “TNF signaling pathway” and “NF-kappa B signaling pathway.” The pro-inflammatory cytokine TNF plays a significant role in apoptosis, cell proliferation, differentiation, necrosis, and cytokine induction (74). Activation of NF-kappa B is thought to guide the transcription of genes associated with inflammation and cell death or survival (75).

The integrated analysis identified DEmiRNA targets to be related to the “mTOR signaling pathway,” “Endocytosis,” and “PI3K-AKT signaling pathway”. PI3K regulates its downstream effector's activity through the AKT/mTOR/p70^S6K^ signaling axis, thereby altering the production of cytokines, and thus could be a potential target for inflammatory diseases (76). Previous studies have also shown that Bta-miR-145 expression was down-regulated in udder tissues after *S. aureus* infection, consistent with our research. It was also found that overexpression of Bta-miR-145 significantly inhibited the proliferation of bMECs, and also reduced the secretion of IL-12 and TNF-α, but increased the secretion of IFN-γ (77). Therefore, the down-regulation of Bta-miR-145 is conducive to the production of IL-12 and TNF-α, thus inducing an immune response.

## Conclusion

In the current study, we characterized the whole transcriptome profiles of bovine mammary epithelial cells infected intracellularly with *S. aureus* by RNA-seq. *S. aureus* invading into bovine mammary epithelial cells can trigger the immune responses, ROS production, and the expression of genes involved in autophagy. These differentially expressed RNAs may be critical in understanding the molecular mechanisms of *S. aureus* to survive in bovine mammary epithelial cells. It thus provides novel insights into the responses to bovine mammary epithelial cells with intracellular *S. aureus*.

## Data Availability Statement

The datasets presented in this study can be found in online repositories. The names of the repository/repositories and accession number(s) can be found in the article/supplementary material.

## Author Contributions

YL and BH: conceptualization. XW and NG: data curation and writing—original draft. XW and FS: formal analysis. XY: investigation. NG and XY: methodology. JL and YL: project administration, writing—review and editing, and supervision. RW: resources. XW: software. LL and RW: validation. MZ: visualization. All authors contributed to the article and approved the submitted version.

## Conflict of Interest

The authors declare that the research was conducted in the absence of any commercial or financial relationships that could be construed as a potential conflict of interest.

## Supplementary Material

Supplementary File 1 Detailed information of DEmRNAs. Differently expressed mRNAs (DEmRNAs) in *S. aureus* infected mammary epithelial cells compared with non-infected mammary epithelial cells.

Supplementary File 2 Detailed information of GO enrichment of DEmRNAs. GO term function classification of DEmRNAs.

Supplementary File 3 Detailed information of DElncRNAs. Differently expressed lncRNAs (DElncRNAs) in *S. aureus* infected mammary epithelial cells compared with non-infected mammary epithelial cells.

Supplementary File 4 Detailed information of GO enrichment of DElncRNAs. GO term function classification of DElncRNAs.

Supplementary File 5 The two gene common target genes from DEmRNA and DElncRNA targets. Intersection of DEmRNA and DElncRNA.

Supplementary File 6 Detailed information of DEmiRNAs. Differently expressed miRNAs (DEmiRNAs) in *S. aureus* infected mammary epithelial cells compared with non-infected mammary epithelial cells.

Supplementary File 7 Detailed information of GO enrichment of DEmiRNAs. GO term function classification of DEmiRNAs.

Supplementary File 8 The gene symbol corresponding to all mRNAs.
